# Medical News and Miscellany

**Published:** 1891-09

**Authors:** 


### Medical News and Miscellany.

Dr. J. M. Dollar has removed from Burlington to Gauze, his former home.

Dr. White's paper in the present issue is most timely and appropriate. What to do with the insane is a problem hard of solution.

The Journal received orders for one hundred copies of August issue more than it was able to supply; hence an extra edition this month.

Do not forget that the Austin District Medical Society holds its regular quarterly meeting in Austin on the 4th Thursday in this month (September), it being the 24th day. An interesting programme has been prepared. See notice in Society Notes.

Dr. J. L. Jones, late of Austin, a graduate of the Medical Department University of Virginia, has located for practice, at Gatesville. The JOURNAL commends Dr. Jones to the profession as a worthy accession to the ranks, and extends to him its best wishes.

We learn with some surprise that Dr. Frank Paschall, Surgeon in-Chief of the Mexican Central Railroad Hospitals at Chihuahua, Mexico, contemplates leaving Mexico to return to San Antonio, where he has already contracted for the erection of a handsome residence; the reason,—to educate his children.

Dr. Geo. Dock the late pathologist of the Texas Medical College has been appointed Professor of Theory of Practice of Medicine in the University of Michigan at Ann Arbor. We venture he is the youngest physician in America to occupy so important a position—but he is a remarkably bright and talented man.

In Diarrhœa, especially of the mucous variety, or where fermentation is taking place in the alimentary tract, salol is a most valuable remedy. Given in five grain doses, either with or without bismuth, in mint water, it acts promptly in checking and changing the character of the discharge and in relieving the tormina which so frequently accompanies the latter condition. Dr. Morris' prescription for salol was given in our June issue.

**Bob-Tailed Directory.**—Under this caption the *Memphis Journal of Medical Science* shows up a fake directory called "The Southern Medical Directory," which purports to have been issued by a firm by name of Gunter, of New Orleans. The book agent evidently "got in his work" on Dr. Rogers; and Rogers is now letting off steam to keep from "bursting." It is, however, a timely warning. The agent got an advertisement out of the Doctor, making promises which he didn't fulfill. Let Texas doctors look-out for the "Bob-tailed Directory" man.

---

**Stories of a Country Doctor.**—Attention is called to the advertisement of this book in the advertising pages.

Dr. King's little book has created a lasting sensation; everybody wants to read it,—and, they will not be disappointed; it is certainly very readable. Its sale has been phenomenal. A large edition was issued a year ago—first edition, and it was rapidly exhausted, selling at $2.50 a copy.

Messrs. Hummel & Parmele, of Philadelphia, have just issued a second edition, revised,—and although handsomely bound in cloth and gilt lettered, and illustrated, they are selling it at *one dollar a copy*. It sells rapidly, as might be expected, at such price.

This JOURNAL has made arrangements whereby the book will be furnished our subscribers at 50 cents per copy, postage paid, if ordered in connection with the JOURNAL; that is, for $2.50 the JOURNAL will be sent one year, and a copy of Dr. King's book will be mailed free: or if the JOURNAL is not wanted send $1 and a copy of the book will be mailed on receipt of price, at this office. If ordered direct from the publishers mention where you saw this notice.

---

**Something New, "Ophthalmic Discs."**—Dr. Casey A. Wood, Pathologist to the Illinois Eye and Ear Infirmary, etc., read a paper before the Chicago Medical Society, July 6, 1891, in which he said that he had been experimenting with a view to find a better, more satisfactory and economical way of introducing the costly alkaloids into the eye; that the conjunctival sac will hold only one drop, and when ten drops of a solution of atropine or other drug are instilled into the eye, nine drops or the greater part of it escape through the nasal duct or with the overflow of tears; and that it is necessary to introduce $\frac{1}{10}$ grain of atropia in that way in order to get the effect of the $\frac{1}{100}$, something to be considered when you come to homatropine which costs seventy-five cents a grain; besides it is not intended to affect the Schneiderian membrane; the escape just mentioned is not desirable, especially where cocaine is used. He finally got Wyeth Bros. to make him some discs after the manner of their compressed hypodermic discs now generally used, and he is pleased with the result. He claims advantages for them over any other mode of eye medication. The disc which will contain the desired dose is introduced by dropping it into the lower lid while the eye is turned upward—by means of a camel hair pencil, to which it readily adheres when slightly moistened. It is not necessary then to waste the 10th of a grain in solution, to get the effect of the $\frac{1}{100}$. Wyeth Bros. have published a list of such discs, and it embraces every drug used in eye practice and in any dose ordinarily used. Send to them for a list, if you are interested.

---

**The Texas Sanitarian.**—THE JOURNAL is in receipt of the "Prospectus of *The Texas Sanitarian*, a Journal of Preventive Medicine and Hygiene," "to be published monthly at Austin, Texas, by the Texas Sanitarian Publishing Company," with Dr. T. J. Bennett, the well known, courteous and efficient Secretary of the Austin District Medical Society as managing editor. Vol. 1, No. 1, a 5000 copy edition, will be issued November 1. The aim of the publication, as announced in prospectus, will be to popularize sanitation, to "make both the profession and the people familiar with the principles of sanitation or preventive medicine." It will aim to interest the teachers of Texas—"those to whose care is entrusted the development of the rising generation in their physical, moral and intellectual life,"—and it is hoped to introduce the work into schools and families.

This proposition must commend itself to all thinking people as eminently sensible and timely; and if it be successfully carried out, no doubt much good will be done. It is time the people were being instructed in these matters,—they do not in many instances know the meaning of the word "hygiene," and as to "State medicine" or "Preventive medicine," they are meaningless to many intelligent people. And that the program announced in this prospectus will be carried out, there is scarcely room to doubt, since, we observe with pleasure the editorial staff numbers such men as State Health Officer Swearingen—the chief of the health department of Texas; President Dr. Wooten, of the

Board of Regents, Texas University; Dr. J. W. McLaughlin, whose writings, in connection with his researches in bacteriology have given him international fame; and Dr. Morris—the Sage of Austin—favorably known as a writer and a physician. *The Sanitarian* claims advantages possessed by no other journal, for obtaining reports of the health officer—vital and mortuary statistics, chemical analyses, etc., and we await with interest the appearance of the initial number. That it will meet a cordial reception and a strong support at the hands of the Texas profession is an assured fact; the opening is splendid, and the JOURNAL predicts it will be ably and acceptably filled. Success to *The Texas Sanitarian*.

The Inter-Continental American Medical Congress.—The committee on permanent organization will meet in St. Louis October 17. Of course, the officers will be selected from this country, though it is expected that South America will be largely represented on the several sections. For the first time in the history of the world the physicians of the Western Hemisphere—from "all the Americas"—will meet for conference. The occasion will be one of great interest, and the meeting at St. Louis, which will be simultaneous with that of the Mississippi Valley Medical, and the Medical Press Association, should be largely attended.

**Dr. Cunningham** writes as follows to a Texas contemporary, anent the subject of reprints:

FORT WORTH, TEXAS, July 28, 1891.

EDITOR HEALTH JOURNAL:—As being of interest to those getting reprints of their papers read at Waco before the T. S. M. A., I send you for publication the following extracts of replies to enquiries sent by me as to price:

AUSTIN, TEXAS, August 7, 1890.

* * * * Reprints will cost you:

| | |
|---|---|
| 100 copies (per page for as many pages as are contained in one copy) | 40 cts. |
| 200 copies as above | 50 " |
| 500 " " | 65 " |

Your paper will make about 10 pages, and therefore it would cost you for 500 copies about *$6.50*. * * * *

EUGENE VON BOECKMANN.

GALVESTON, TEXAS, July 13, 1891.

* * * * 200 reprints *without* cover and title page will cost you $5.00; with cover, etc., *$8.00*. * * *

H. A. WEST, Sec. T. S. M. A.

My paper read at Waco contained about 1300 words, making, therefore, about 3½ pages the size of those of proceedings of 1890. Von Boeckmann's charges *included cover and title page*. It was a 10-page paper. I had 500 copies, with cover and title page, sent me for $6.50.

Let us now place the charges side by side and look at them as we would look at two horses offered us for purchase:

| AUSTIN'S OFFER. | | GALVESTON'S OFFER. | |
|---|---|---|---|
| 200 copies reprints, 3½ pages, at 50c. per page | $1.75 | 200 copies reprints, 3½ pages, at $2.2-7 per page | $8.00 |
| 200 copies reprints, 10 pages, at 50c. per page | $5.00 | 200 copies reprints, 10 pages, at $2.2-7 per page | $22.85 |
| Look at this picture. | | Now look at this. | |

This beats "before taking" and "after taking."

Respectfully,

J. L. CUNNINGHAM.

---

A "**Canser Doctor.**"—A subscriber sends the JOURNAL the following as a specimen of the kind of "doctors" infesting his section. We give a *verbatim* copy, changing names to prevent identification of the victim. The gentleman addressed, and whom we call "Smith," is a patient of our correspondent, a leading physician of —— county. The envelope is addressed as follows:

"John smith
Kent naler
co txas"

"August 29, 1891.

" wallis po collin co
txas

"Mister John smith

"Sir i am informed By a frend of yours that you have a canser i am a canser Doctor i doctor By the insurence i will cure you fur a fare Reasoneable price if you want Mee to cure you wright Mee at once what cind of canser is yours is it Driping Much tell me all a Bout it i i would have to Bord with you till it got well fur a comon canser i charg $100 one hondred dollars and Board and washing one half of it when the canser is taken out the other half when the place is cured up tell Mee whir yours is wright me imeadely if you want Mee to take it out and tell Mee if you have Ever had it cut or Burnt By Doctors

"J F ——

"to John smith if i have not go the right name of person will you pleas take this too him and oblige

"James F ——"

Talk about suppressing quackery—" Not that any body knows of." The " Canser Doctor" flourisheth like a green bay tree—perennial and luxuriant. He is abroad in the land, and the law-makers know it.

